# Hetero-Aggregation of Nanoplastics with Freshwater Algae and the Toxicological Consequences: The Role of Extracellular Polymeric Substances

**DOI:** 10.3390/toxics13110980

**Published:** 2025-11-14

**Authors:** Jiannan Ding, Jiaxin Yang, Xiaojun Song, Shuo Liu, Zhenguo Wang, Hua Zou

**Affiliations:** 1 School of Environment & Ecology, Jiangnan University, Wuxi 214122, China; djn@jiangnan.edu.cn (J.D.); 6231403018@stu.jiangnan.edu.cn (J.Y.); 6243502076@stu.jiangnan.edu.cn (S.L.); 2310659@tongji.edu.cn (Z.W.); 2 Jiangsu Collaborative Innovation Center of Technology and Material of Water Treatment, Suzhou 215009, China; 3 Biomass Energy and Biological Carbon Reduction Engineering Center of Jiangsu Province, Wuxi 214122, China

**Keywords:** polystyrene, polylactic acid, tightly bound EPS, loosely bound EPS, integrated biomarker response

## Abstract

The presence of nanoplastics (NPs) in freshwater environments has received increasing attention in recent years. However, the hetero-aggregation behaviors of NPs with the co-existing algae and the influence on NP toxicity, especially the potential role of extracellular polymeric substances (EPS) during the entire process, are poorly understood. In this study, the hetero-aggregations of polystyrene (PS) and polylactic acid (PLA) NPs with *Chlorella vulgaris*, along with their toxicological consequences, were investigated in EPS-containing and EPS-free conditions. The results in the 12 h settling experiments showed that the ΔOD_reduced_ values ranged from 0.33 to 0.74, and the PS NPs exhibited higher aggregation efficiency with algae than the PLA NPs, which was inconsistent with previous microplastic studies and the Derjaguin–Landau–Verwey–Overbeek calculations. This can be attributed to the unique properties of NPs and the mediating effects of tightly bound and loosely bound EPS during the formation of stable heteropolymers. In the 96 h toxicological experiments, various endpoints for algal growth inhibition, pigment synthesis disturbance, cell membrane damage, and oxidative stress were measured. Both the ΔOD_reduced_ values and integrated biomarker responses were positively associated with membrane damage and superoxide dismutase activity, demonstrating a view that the hetero-aggregation behavior could affect the membrane integrity and oxidative stress of algal cells, and exacerbate the toxicity of NPs on algae. The present study underscores the material-specific uniqueness of NPs in interactions with freshwater algae. Further studies are needed to broaden our knowledge of the hetero-aggregation behaviors and toxicological effects of NPs.

## 1. Introduction

Plastics in aquatic environments may undergo various weathering processes and fragment into smaller particles [1]. Nanoplastics (NPs) are defined as plastic particles <1 μm in diameter [2]. They can originate from the weathering of larger plastics or can be synthesized and used as exfoliants in personal care products. Due to their unique physicochemical nanoscale properties, NPs may exhibit environmental behaviors and toxicities that are distinct from micro- (1–5000 μm) and macro-plastics (>5000 μm) [3]. However, the environmental behavior and toxicity of NPs have been less explored than those of micro- and macro-plastics [4].

Natural aquatic systems comprise a diverse array of colloidal particles, including natural organic matter, biological colloids, and inorganic colloids [5]. Due to their heterogeneity, NPs may undergo homo- or hetero-aggregation upon release into aquatic environments [4]. In saltwater environments (e.g., estuaries and the ocean), NPs are likely to form homo-aggregates according to the Derjaguin–Landau–Verwey–Overbeek (DLVO) theory [6]. However, NPs in freshwater environments (e.g., lakes and rivers) are struggle to form homo-aggregates, because the ionic strength of water is probably lower than the critical coagulation concentration [4,7]. As a result, NPs in freshwater environments may tend to form hetero-agglomerates in most cases. Algae are common biological colloids in aquatic ecosystems, and could suffer from nanoparticle contamination by forming hetero-aggregates [8]. Some studies have demonstrated that manufactured metallic nanoparticles are prone to aggregate with algal cells [9,10,11]. Although NPs and metallic nanoparticles share overlapping size ranges, their environmental behaviors and fates can be radically different when considering other characteristics, such as surface chemistry, density, and morphology [3]. However, research on hetero-aggregation between NPs and freshwater algae is still in its infancy.

To date, most studies about the hetero-aggregation of plastic particles with algae have focused on microplastics. Lagarde et al. [12] preliminarily discovered the presence of hetero-aggregates between polypropylene (PP) microplastics (>400 µm) and *Chlamydomas reinhardtii* through microscopic observation. As research has progressed, the role of algae-derived extracellular polymeric substances (EPS) in microplastic–algae interactions has received increasing attention. The EPS matrix is composed of biopolymers, including polysaccharides, proteins, and nucleic acids, which can interact with other polymers in the surrounding environment [13]. Generally, the viscous EPS is considered favorable for hetero-aggregation during the interaction between microplastics and algae [14]. Cunha et al. [15] investigated the hetero-aggregations of poly(methyl methacrylate) (PMMA) and polystyrene (PS) microplastics with two marine (Tetraselmis sp. and Gloeocapsa sp.) and two freshwater algae (Microcystis panniformis and Scenedesmus sp.). Those authors found that the hetero-aggregations were depended both on plastic type and on the content and viscosity of EPS. Su et al. [16] compared the hetero-aggregations of the fuel-based (PS) and bio-based (polylactic acid, PLA) microplastics with *Chlorella vulgaris* and found that the formation of microplastic–algae aggregates was positively associated with EPS content. For NPs, the aggregation mechanism could be different from those of microplastics, owing to their unique size-emergent properties [17]. Moreover, the role of EPS in the hetero-aggregation behaviors of NPs with algae could also be varied. According to their ability to bind to the cell surface, EPS can be characterized as either tightly bound (TB) or extending outward from the cell surface towards the surrounding environment as a loosely bound (LB) [18]. Due to their distinct constituents and properties, the effects of the two types of EPS have been reported to be different, or even contradictory, on various behaviors of coexisting substances, such as sludge aggregation [19], antibiotic adsorption [20], and antibiotic-resistance gene transfer [21]. In comparison, the specific roles of the different EPS components during hetero-aggregation between NPs and algae remain ambiguous.

In addition to the biological colloids, algae play an important role as primary producers in freshwater ecosystems. Thus, elucidating the aquatic toxicity to algae is indispensable for accurately assessing the ecological risks of NPs. As a direct interfacial interaction, hetero-aggregation with algal cells is an important prerequisite for the toxicity of NPs [22]. Along with direct contact, NPs can enter algal cells through endocytosis or internalization, inducing adverse effects on algae [23]. Even if NPs do not enter algal cells, they may still trigger biological toxicity through indirect mechanisms, such as shading effects, which lead to light attenuation and reduce the energy available for algal photosynthesis [24]. Previous studies have documented the toxicity of NPs to algae by assessing their growth, photosynthetic system activity, cell viability, and indicators of antioxidant defense and oxidative damage [22,25]. However, the potential mediating effects of hetero-aggregation behavior and the influence of EPS are often overlooked. This may lead to a misunderstanding of the environmental risks posed by NPs in aquatic ecosystems. Zhao et al. [26] found that during nano-ZnO exposure, EPS from *C. vulgaris* may play a significant role in the aggregation with metallic nanoparticles, minimizing direct particle interactions with the cell membrane. However, Zhang et al. [27] indicated that EPS could attract and retain more PS NPs around algae (*Thalassiosira pseudonana*), producing more reactive oxygen species (ROS). EPS could play a complicated role in NP toxicity on algae, and this may be a result of its diverse components with various natures, such as TB- and LB-EPS fractions. Hence, further investigations into this issue are urgently required.

The objectives of this study were to (1) understand the hetero-aggregation behavior of NPs with algae and its influence on NP toxicity and (2) explore the mediation effects of EPS during the entire interaction process. In this study, *C. vulgaris* was used as a model alga. Two types of nano-polymers, namely the fuel-based PS NPs and the bio-based PLA NPs, were used as the target NPs. Our previous study and other reports have demonstrated that large amounts of PS and PLA NPs can be generated from plastic products, and their environmental fates and potential risks require immediate attention [28,29,30,31]. To assess the aggregation interactions between the NPs and *C. vulgaris*, settling experiments were conducted, the interaction energies involved in the hetero-aggregation process were analyzed according to the DLVO theory, and the composition of TB- and LB-EPS fractions was characterized using a three-dimensional excitation emission matrix (3D-EEM). *C. vulgaris* was then exposed to PS and PLA NPs at two different concentrations (1 and 10 mg/L) for 96 h. The toxicological consequences, including algal growth inhibition, pigment synthesis disturbance, cell membrane damage, and oxidative stress, were investigated. Furthermore, using the cation exchange resin method, algal cells with and without EPS were obtained, and the role of EPS during the interactions between NPs and algae was comparatively analyzed. The findings of this study will broaden our comprehension of the ecological risks of NPs in freshwater environments.

## 2. Materials and Methods

### 2.1. NP Preparation

PS and PLA spheres with nominal particle sizes of 20 nm were purchased from Goose Technology Company (Tianjin, China) and Ruixi Biological Company (Shaanxi, China), respectively. According to the manufacturers’ information, no antimicrobials or surfactants were added during the synthesis process of the PS and PLA spheres. For each plastic, three transmission electron microscope (TEM) images were recorded, and twenty particles were randomly selected in each image. In total, the grain size distribution of 60 particles for each plastic was analyzed using the ParticleSizer tool in ImageJ (v1.54) software based on the method of Zhang and Wang [32]. The zeta potential was measured by a nanoparticle zeta potential meter (Litesizer 500, Anton Paar, Graz, Austria) equipped with a 658 nm wavelength laser source, and the applied voltage was kept at 200 V. The samples were allowed to rest for 2 h at room temperature before measurement and were kept in omega-shaped plastic cuvettes of 700 μL volume. The chemical properties were characterized by a Fourier-transform infrared spectrometer (FTIR; IRTracer-100, Shimadzu, Kyoto, Japan). FTIR measurements were performed in attenuated total reflection (ATR) mode, collecting spectra from 600 to 3600 cm^−1^ at a resolution of 2 cm^−1^ over 32 scans. Before the experiment, the NP stock suspension was sonicated in an ultrasonic bath (40 kHz) for 30 min to disperse the particles.

### 2.2. Algae Preparation

*C. vulgaris* FACHB-2338, sourced from the Freshwater Algae Culture Collection at the Institute of Hydrobiology (FACHB, Wuhan, China), was cultivated in autoclaved standard blue-green (BG-11) medium (pH 7.0). The constituents of the culture medium are listed in Tables S1 and S2. Algae were inoculated into sterilized Erlenmeyer flasks on an aseptic bench (SW-CJ-2FD, Boxun, Shanghai, China) and placed in an illumination incubator (SPX-250B-Z, Boxun, Shanghai, China). The incubator was set to maintain a light intensity of 6000 lux, a temperature of 25 ± 1 °C, and a light/dark cycle of 12 h each. The Erlenmeyer flasks were manually shaken three times daily to inhibit the clumping of algal cells. The zeta potential and size of algae cells can be found in Table S3.

Algal cells in the exponential growth phase (days 6−15) were harvested by centrifugation at 600× *g* for a duration of 15 min at 25 °C, followed by two washes with Milli-Q water. The cells with algae-derived EPS were denoted as EPS-containing, i.e., EPS-C. Algal cells without EPS were obtained using the cation-exchange resin method [33]. Briefly, pre-cultured *C. vulgaris* (days 6−15) were collected by centrifugation at 600× *g* for 15 min at 4 °C. Following two washes with Milli-Q water, the algae were combined with cation-exchange resin (Dowex Marathon C, DuPont, Wilmington, DE, USA) in a mass ratio of 1:2.5 and stirred at 600 rpm for a duration of 1.5 h. After the mixing process, the mixture was subjected to centrifugation at 9600× *g* for 20 min at a temperature of 4 °C. The remaining algae were collected and denoted as EPS-free, i.e., EPS-F. Representative TEM observations of EPS-C and EPS-F cells are presented in Figure S1, suggesting that the two kinds of cells have been successfully obtained.

### 2.3. Analysis of Hetero-Aggregation Between NPs and Algae

#### 2.3.1. Settling Experiments

Settling experiments were conducted to understand the hetero-aggregation behavior of NPs after contacting with the algal cells. Overall, the settling experiments consisted of individual and co-settling experiments. For individual settling experiments, 50 mL suspensions of two types of NPs (i.e., PS and PLA) and two types of algal cells (i.e., EPS-C and EPS-F) were placed separately in glass tubes for 12 h. For co-settling experiments, 50 mL mixed suspensions of PS NPs + EPS-C algae, PS NPs + EPS-F algae, PLA NPs + EPS-C algae, and PLA NPs + EPS-F algae were added into glass tubes, and the suspensions were allowed to stand for 12 h. In each treatment, the density of algal cells was 1 × 10^6^ cells/mL, and the concentration of NPs was 10 mg/L. Each treatment was performed in triplicate. Before the experiments, the suspensions were sonicated at 40 kHz for 5 min at room temperature (25 °C) to achieve uniform dispersion. As observed using a microscope, the sonication did not change the morphological and structural integrity of algal cells (Figure S2). Then, the suspensions were set aside to settle naturally. During settling, 2.5 mL of suspension was taken at 0.5, 1, 1.5, 2, 3, 4, 5, 6, 7, 8, 10, and 12 h, and placed in 3.5 mL glass colorimetric dishes to measure absorbance. The absorbance measurements were performed by using a UV-2600 spectrophotometer (Shimadzu, Japan) at 680 nm. The normalized individual and co-settling curves were plotted with the normalized (A/A0) against the settling time (t), while the normalized additive-settling curves depicted the sum of the independent absorbances of NPs and algal cells related to settling time. All the settling curves were plotted and aligned with Equation (1):

*y* = *OD_plateau_* + *OD_reduced_ e^−νt^* (1)

where OD_plateau_ represents the optical density at the plateau of the settling curve, OD_reduced_ is the decreased optical density owing to hetero-aggregation at the plateau of the settling curve, *v* (OD/min) is the settling rate, and *t* (min) is the settling time. The ΔOD_reduced_ parameter is calculated by subtracting the OD_reduced_ of the sum-settling curve from the OD_reduced_ of the co-settling curve. The hetero-aggregation could occur when ΔOD_reduced_ > 0. Contrarily, a zero or negative ΔOD_reduced_ value indicates negligible or non-existent hetero-aggregation.

#### 2.3.2. DLVO Theory Analysis

The DLVO theory was utilized to assess the colloidal interaction energies between nanoparticles and algal cells. It can theoretically forecast the hetero-aggregation behavior of aqueous dispersions and determine the total interaction energy as a function of the separation distance [34]. In this study, the DLVO energy was calculated as a combination of the van der Waals attractive and electrostatic interaction energies. The DLVO interaction potentials of NPs and algae were calculated using Equations (2)−(6).

Total interaction energy of DLVO:

*V_T_* (*h*) = *V_A_* (*h*) + *V_R_* (*h*) (2)

The Van der Waals force calculation:

(3) $$V_{A}\left(h\right)=-\frac{Ar_{1}r_{2}}{6h(r_{1}+r_{2})}{\left[1-\frac{5.32h}{\lambda }ln(1+\frac{\lambda }{5.32h})\right]}^{-1}$$

where *A* is Hamaker’s constant, with a value of 7.5 × 10^−21^ J used in this study; *r*_1_ and *r*_2_ are the radii of the NPs and the algal cells, respectively; *h* is the distance between the algal cells and the NPs; and *λ* is the characteristic wavelength of the interaction.

Electrostatic repulsion calculation between algae and NPs:

(4) $$V_{R}\left(h\right)=\frac{2\pi r_{1}r_{2}n_{\infty }kT}{\left(r_{1}+r_{2}\right)\kappa^{2}}(\psi_{1}^{2}+\psi_{2}^{2})\left\{\frac{2\psi_{1}\psi_{2}}{\psi_{1}^{2}+\psi_{2}^{2}}\ln\left[\frac{1+exp(-\kappa h)}{1-exp(-\kappa h)}\right]+ln\left[1-exp(-2\kappa h)\right]\right\}$$

Here, *n*_∞_ is the ion number density in solution; *κ* is Boltzmann’s constant, 1.38 × 10^−23^ J K^−1^; *T* is the thermodynamic temperature; *ψ*_1_ and *ψ*_2_ are the differential surface potentials of NPs and algal cells, respectively; and *κ* is the Debye–Huckel derivative, calculated as follows:

(5) $$\kappa =3.28\times \sqrt{I}\times 10^{9}$$

where *I* is the ionic strength of the background solution.

(6) $$\psi =\frac{ze\zeta }{kT}$$

Here, *z* is the ionic valence of the solution; *e* is the electronic charge, 1.60 × 10^−19^ C; and *ζ* is the experimentally measured zeta potential.

#### 2.3.3. EPS Analysis

After the co-settling experiments, the physicochemical characteristics of EPS from *C. vulgaris* under NPs stress were examined. Two categories of EPS (i.e., LB- and TB-EPS) were distinguished based on the method of Li et al. [21], which relies on sequential extraction using physicochemical treatments of different intensities. LB-EPS is the fraction recovered by gentle centrifugation, representing polymers loosely associated with the cell surface, and TB-EPS is the fraction requiring stronger extraction conditions (e.g., thermal treatment at 60 °C) to be released, representing the tightly bound polymeric matrix. Specifically, 10 mL algae suspension in each treatment was collected and centrifuged at 1000× *g* for 15 min at a temperature of 4 °C. The supernatant was then collected and the EPS remaining in the supernatant was classified as LB-EPS. The algal pellets were gathered and redissolved with 0.6% NaCl to 10 mL to maintain the osmotic equilibrium and to prevent cell lysis. Subsequently, the suspension was incubated at 60 °C in a water bath for 30 min. After cooling down to room temperature, the suspension underwent centrifugation at 4 °C for 15 min at a speed of 6700× *g*, after which the supernatant was gathered and the EPS remaining in the supernatant was classified as TB-EPS.

All the collected supernatants were filtered through a 0.45 μm acetate fiber membrane and scanned by 3D-EEM fluorescence spectroscopy (F-7000, HITACHI, Tokyo, Japan). Emission scans ranged from 200 to 500 nm in 5 nm steps, with excitation wavelengths covering 200–550 nm, and stepping 5 nm each. The photomultiplier voltage was set at 600 V. The spectral scanning speed was kept at 12,000 nm/min, with a response time of 0.002 s. The spectrum of ultrapure water was measured as a blank to minimize Raman scattering from water and lower the background noise. The EEM spectra are represented by the elliptical contours.

#### 2.3.4. Algal Ultrastructure Analysis

Alterations in algal ultrastructure after 12 h of co-settling were observed using a transmission electron microscope (TEM; JEM-1200EX, JEOL, Tokyo, Japan). Algae preserved with glutaraldehyde underwent triple cleansing in a 0.1 M solution of PBS buffer, calibrated to a pH level of 7.4. Following this, they were fixed in 1% osmic acid at 4 °C for 4 h in the dark. The algae were then dehydrated in alcohol, embedded in epoxy resin, sectioned into slices, and stained using lead citrate according to the method of Sadiq et al. [35].

### 2.4. NP Toxicity Experiments

#### 2.4.1. NP Exposures

The NP exposure experiments consisted of 8 treatments [2 types of NPs (i.e., PS and PLA) × 2 concentration levels of NPs (i.e., 1 and 10 mg/L) × 2 types of algal cells (i.e., EPS-C and EPS-F)], as well as 2 controls that contained only EPS-C or EPS-F algal cells without NPs. The exposure time was 96 h, and each treatment was performed in triplicate. Specifically, pre-cultured algae were added to 250 mL conical flasks containing 100 mL of BG-11 medium. The initial density of the algae in each conical flask was 1 × 10^6^ cells/mL. The exposures were conducted in an illumination incubator (SPX-250B-Z, Boxun, Shanghai, China). The incubator was set to maintain a light intensity of 6000 lux, a temperature of 25 ± 1 °C, and a 12 h/12 h light/dark cycle. NPs were added to conical flasks at environmentally relevant concentrations of 1 and 10 mg/L, respectively. Descriptions about the environmentally relevant concentrations of the PS and PLA NPs can be found in Text S1 in the Supplementary Materials.

#### 2.4.2. Determination of Algal Growth

During the exposures, the cell densities (cells/mL) at 0, 24, 48, 72, and 96 h were assessed using a hemocytometer. To ensure accurate counting, algal cells were shaken and added dropwise to the hemocytometer. Each sample was counted twice. The algae density was calculated according to Equation (7), and growth inhibition was calculated using Equation (8):

(7) $$\mathrm{Algae}\;\mathrm{density}\;(\mathrm{cells}/\mathrm{mL})=\;\frac{x}{80}\times 400\times 10,000$$

(8) $$\mathrm{Inhibition}\;(\%)=\frac{C_{ct}-C_{et}}{C_{ct}}\times 100$$

where x is the number of algal cells in the hemocytometer and *C*_ct_ and *C*_et_ are the cell count densities of the control and experimental treatments, respectively.

#### 2.4.3. Determination of Pigment Synthesis

Chlorophyll a and carotenoids levels were measured at various time intervals (0, 24, 48, 72, and 96 h) following the procedure described by Su et al. [36], with slight modifications. First, a 5.0 mL suspension of algae was centrifuged at 6700× *g* for a duration of 10 min at a temperature of 4 °C. Following the removal of the supernatant, 5.0 mL of 90% ethanol was introduced to facilitate pigment extraction, with the mixture being incubated at 60 °C in a water bath for 20 min. Once the samples had cooled to room temperature, they were centrifuged again at 6700× *g* for 10 min at 4 °C. The supernatant was gathered for the determination of pigment contents using a UV-spectrophotometer (UV2600, Shimadzu, Japan). The absorbance of the supernatant was measured in triplicate at 665, 652, and 470 nm. To evaluate potential interference from the NPs with the spectrometry-based assays, we prepared NP-free algal medium and algal medium containing 10 mg/L of PS or PLA NPs, and processed them identically to the actual samples, including all pretreatment steps. The absorbance readings at 665, 652, and 470 nm revealed no significant differences among the NP-free, PS NP-added, and PLA NP-added samples, indicating no substantial interference from the NPs themselves (Table S4). The Chlorophyll a, Chlorophyll b, and carotenoids contents were determined using the following equations:

*Chlorophyll a* (mg/L) = 16.82 × *A*_665_ − 9.28 × *A*_652_ (9)

*Chlorophyll b* (mg/L) = 36.92 × *A*_652_ − 16.54 × *A*_665_ (10)

(11) $$\text{Carotenoids}\;(\mathrm{mg}/L)=\frac{1000A_{470}-1.91\;\mathrm{Chlophyll}\;\;a-\;95.15\;\mathrm{Chlophyll}\;b}{225}$$

where *A* is the absorbance of the supernatant at a specific wavelength.

#### 2.4.4. Determination of Algal Membrane Integrity

The membrane integrity of the cells was analyzed using propidium iodide (PI) staining [37]. When the cell membrane of the algae is compromised, propidium iodide infiltrates these cells, staining the nucleic acids and producing an intense red fluorescence. Therefore, propidium iodide can be used to assess algal cell membrane integrity. After 96 h of exposure, the algal suspensions were centrifuged at 1600× *g* for 30 min. After discarding the overlying supernatant, the algal cells were cleaned twice in 0.1 M PBS buffer (pH 7.4). Propidium iodide (Sigma-Aldrich, Shanghai, China) was then introduced into the algal cultures at a final concentration of 5 mg/L. After incubation in darkness for 15 min at room temperature (25 °C), the fluorescence intensity was quantified within the FL3 channel (533/30 nm) using a Flow Cytometer (CytoFLEX S, Beckman Coulter, Brea, CA, USA).

#### 2.4.5. Determination of Oxidative Stress

After 96 h of exposure, the antioxidant response of *C. vulgaris* was analyzed. First, the microalgal cells were centrifuged for 10 min at 6700× *g* to remove the overlying supernatant. Appropriate amounts of extract solution (e.g., 5 × 10^6^ cells in 1 mL extract solution) were added. Subsequently, the cells were lysed using an ultrasonic cell crusher set to a power level of 25%. This process involved ultrasonication for 3 s, interspersed with 10 s intervals, and was repeated 30 times while maintained in an ice bath. Following this, centrifugation was performed at 6700× *g* for a duration of 10 min. The supernatants were treated using the Diagnostic Reagent Kits (Comin Biotechnology Co., Ltd., Suzhou, China) to assess the activity of superoxide dismutase (SOD; WST-8 method) and the content of malondialdehyde (MDA; thiobarbituric acid method), in accordance with the manufacturer’s instructions. The biomarker status was evaluated using a UV-visible spectrophotometer (P7; Mapada Instrument, Shanghai, China). The SOD activity was reported as U/10^6^ cells and was measured at a wavelength of 450 nm. The MDA content was expressed as nmol/10^6^ cells and determined at wavelengths of 532 nm and 600 nm. Following the same approach as for pigment determination, dedicated interference tests of NPs with spectrometry-based assays were conducted. The results showed no significant differences in the absorbance readings at 450, 532, and 600 nm among the NP-free, PS NP-added, and PLA NP-added algal media (Table S4).

#### 2.4.6. Integrated Biomarker Response Analysis

The integrated biomarker response (IBR) was calculated according to Beliaeff and Burgeot [38] and Sanchez et al. [39]. The greater the IBR index, the more modulated the biomarkers are and the more the organism is affected [40]. The IBR was calculated using Equations (12)–(14):

*Y_i_* = log (*X_i_*/*X*_0_) (12)

Here, *Y*_i_ is the standardized value, *X*_i_ is the mean value of each biomarker indicator, and *X*_0_ is the control biomarker indicator.

*Z_i_* = (*Y_i_* − *μ*)/*s* (13)

Here, *Z*_i_ is the mean of standardized biomarker response, *μ* is the mean value of *Y*_i_, and *s* is the standard deviation of *Y*_i_.

*A* = *Z_i_* − *Z*_0_ (14)

Here, *A* is the biomarker deviation index and *Z*_0_ is the mean of the reference biomarker data.

### 2.5. Statistical Analysis

Data were visualized utilizing Origin 2021 (version 9.8.0.200) and reported as the mean ± standard deviation (SD) unless specified otherwise. Statistical evaluations were conducted using one-way or two-way analysis of variance (ANOVA), followed by a least significant difference (LSD) test. These statistical analyses were carried out with SPSS software (version 26.0; IBM SPSS Inc., New York, NY, USA). The Mantel test based on the Pearson method was carried out using R (version 4.5.1) to elucidate the correlations between hetero-aggregation and toxicological endpoints. A probability level of *p* < 0.05 was established as the threshold for statistical significance.

## 3. Results and Discussion

### 3.1. Hetero-Aggregations Between NPs and Algae in Settling Experiments

**Figure 1:**
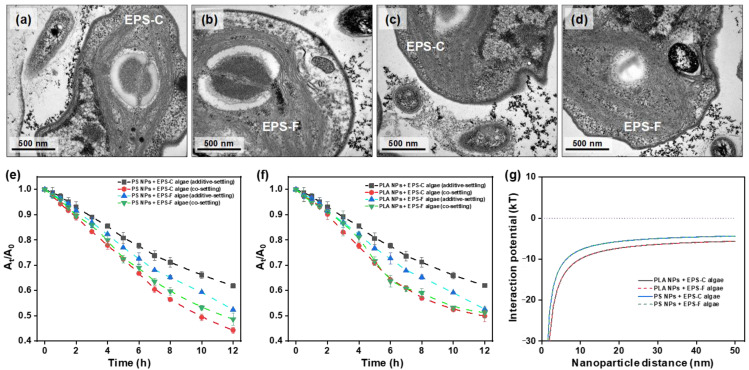
Representative TEM observations of PLA NPs encountering the EPS-C (**a**) and EPS-F (**b**) cells of *Chlorella vulgaris*, and PS NPs encountering EPS-C (**c**) and EPS-F (**d**) algae cells. Additive- and co-settling of *C. vulgaris* with PLA (**e**) and PS NPs (**f**) in different EPS conditions. Total interaction potential energies of PLA and PS NPs with *C. vulgaris* based on DLVO calculations (**g**).

The particle sizes of PS and PLA NPs were 20.4 ± 5.21 and 21.7 ± 4.66 nm, respectively (Figure S3). The zeta potentials of PS and PLA suspended in deionized water were −2.87 ± 0.68 and −1.80 ± 0.45 mV, respectively. The polydispersity indices of PS and PLA NPs were 0.16 and 0.18, respectively, indicating that the two types of NPs were evenly distributed. The FTIR spectra of PS NPs showed typical infrared absorption peaks at 680, 757, 1454, 1490, 1602, 2922, 3025, and 3435 cm^−1^; PLA NP spectra showed absorption peaks at 693, 755, 870, 1092, 1188, 1757, 3000, and 3430 cm^−1^ (Figure S4). Overall, the results indicated that the spheres possessed typical characteristics of PS and PLA NPs. Following the 12 h co-settling, TEM was applied to directly observe the potential formation of the NP-algal hetero-aggregates. As illustrated in Figure 1a–d, a number of PLA and PS NPs were observed to be in contact with and around the algae, indicating the occurrence of hetero-aggregation. In previous studies, the hetero-aggregation of PS NPs with algal cells (*Chlamydomonas reinhardtii*) has been observed by scanning electron microscopy [41]. The TEM images in our study further corroborated the capability of PS NPs to aggregate with freshwater algae through ultrastructure observation and demonstrated that PLA NPs also have hetero-aggregation potential. When the NPs and algae coexisted, they tended to form primary hetero-aggregates. The primary hetero-aggregates could attach to other NP particles in the presence of EPS, and formed larger clusters. Moreover, in the clusters from the TEM images, some cells appeared to have lost their spherical morphology, and the intrusion of NPs into algal cells was observed, implying potential stress effects and toxicity of NPs on algal cells.

To quantitatively describe the hetero-aggregation process, the settling curves and ΔOD_reduced_ values were analyzed in the settling experiments. The co- and additive-settling curves of NPs and algae are shown in Figure 1e,f, and the calculated ΔOD_reduced_ values are listed in Table 1. The individual settling curves for the NPs and algae by themselves can be found in Figure S5 in the Supplementary Materials. Regardless of the type of NPs (i.e., PLA or PS) and EPS condition of algal cells (i.e., EPS-C or EPS-F), there were apparent discrepancies between the co- and additive-settling curves. All the co-settling curves were below the additive-settling curves, and the ΔOD_reduced_ values were positive. Specifically, following the 12 h settling period, the ΔOD_reduced_ values in PS NP treatments were 0.74 and 0.53 for EPS-C and EPS-F algal cells, respectively, and those of PLA NP treatments were 0.33 and 0.49, respectively. The results indicated that there were hetero-aggregations between the NPs and algal cells across the treatments, which were consistent with the TEM observations. The occurrence of hetero-aggregation between NPs and algal cells was also supported by the interaction energy profiles calculated based on the DLVO theory (Figure 1g). The DLVO calculation showed that there were no primary energy barriers in the presence of NPs, which enhanced the interaction with algal cells and made hetero-aggregation more likely to occur [42]. In addition, due to the low electrostatic repulsion energies and negative total interaction potentials, the hetero-aggregation between NPs and algae was predominantly governed by van der Waals attractions, while electrostatic repulsion was insufficient to counteract this process [43].

Comparing the results in the settling experiments between PS and PLA NPs treatments, it can be found that PS NP treatments displayed larger ΔOD_reduced_ values than the PLA NP treatments (Table 1). The result suggested that PS NPs had higher aggregation efficiencies with *C. vulgaris* than PLA NPs. This was inconsistent with the result from the DLVO calculations, in which higher total interaction potentials in the PLA NP treatments than those in the PS NP treatments were displayed (Figure 1g). Apart from the theoretical DLVO considerations, Su et al. [16] provided an empirical example comparing the hetero-aggregation between PS and PLA microplastics with *C. vulgaris*, in which PLA microplastics showed stronger aggregation with *C. vulgaris* than PS microplastics under optical microscope and scanning electron microscopy observations. The discrepancy in hetero-aggregation patterns between NPs and microplastics may be attributed to various factors. For microplastics, the transport was driven by gravitational settling. In this scenario, due to their polar surface functional groups, PLA microplastics would be more readily entrapped by *C. vulgaris* than PS microplastics [44]. Given the nano-size effects, the transport of NPs in water was mainly driven by Brownian motion and diffusion. PS NPs with higher hydrophobicity would be more likely to collide with and adhere to algal cells, while PLA NPs with hydrophilic surfaces may experience repulsive interactions after collisions, partially counteracting the aggregation effect [3]. Collectively, there must be further considerations of the nano-size effects in the hetero-aggregation between NPs and algal cells, apart from theoretical calculation and analogy of empirical examples for microplastics.

### 3.2. Influence of EPS on Hetero-Aggregations Between NPs and Algae

**Figure 2:**
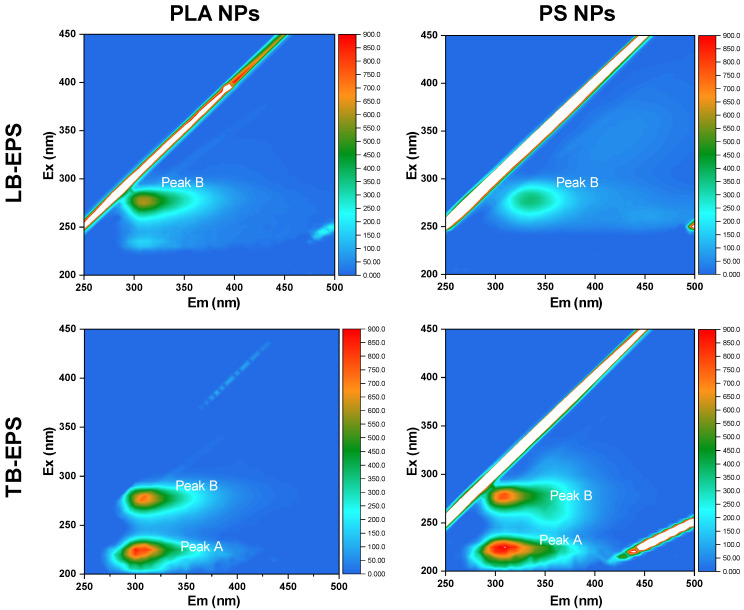
The variations in physicochemical compositions of algal LB- and TB-EPS after 12 h co-settling under different NP conditions. Two fluorescence peaks, peak A and peak B, could be identified and assigned to tyrosine-like and tryptophan-like substances, respectively.

Comparing the results in the settling experiments between EPS-C and EPS-F algal cells, we found that the PS and PLA NPs treatments showed different variation patterns in ΔOD_reduced_ values. In the PS NPs treatments, the ΔOD_reduced_ value with EPS-C condition was higher than that with the EPS-F condition, while in the PLA NPs treatments, the EPS-C algal cells showed a lower ΔOD_reduced_ value than EPS-F algal cells (Table 1). The results suggested that the algal-derived EPS promoted the hetero-aggregation of algal cells with PS NPs, but was unfavorable for that of PLA NPs. The discrepancy could be related to the variations in EPS compositions during the hetero-aggregation. In this regard, fractions of LB- and TB-EPS in the EPS-F algae treatments were further investigated by 3D-EEM spectra after 12 h of co-settling experiments. As shown in Figure 2, two prominent fluorescence peaks (peak A and B) were detected in the TB-EPS, while in the LB-EPS, only peak B was clearly observed. Peak A appeared around 225–230 (λ_Ex_) and 310–320 nm (λ_Em_), and peak B was around 280 (λ_Ex_) and 305–310 nm (λ_Em_). Peak A and B were assigned to tyrosine- and tryptophan-like substances, respectively [45]. Regardless of the NP types, the fluorescence intensities of TB-EPS were higher than those of LB-EPS. The results indicated that NP stress triggered an algal protective mechanism by stimulating EPS secretion, and that the TB-EPS secretion was more active than that of LB-EPS under the NP stress. When comparing the fluorescence intensities of EPS between PS and PLA NPs treatments, it can be found that the EPS secretion depended to some extent on polymer types. Specifically, the fluorescence intensity of LB-EPS in the PLA NPs treatment was higher than that in the PS NPs, while for TB-EPS, its secretion was stronger in the PS NPs treatment. This may be because the aromatic PS NPs triggered stronger stress effects in *C. vulgaris* than the bio-based PLA NPs, the TB-EPS was secreted preferentially, and the secretion of LB-EPS was suppressed accordingly.

Linking the variations in EPS secretion to hetero-aggregation outcomes, it has been reported that the protein constituents of EPS, such as tyrosine- and tryptophan-like substances, contribute to the hydrophobic domains and enhance the ‘stickiness’ of EPS, meaning that EPS plays an essential role in adhesion and is effective in promoting aggregation [26,27,46,47]. For microplastics, Cunha et al. [15] found that *Gloeocapsa* sp. had the potential to colonize PS microplastics through hetero-aggregation, where EPS could accelerate the aggregation process. Cheng and Wang [48] indicated that algae-derived EPS favored the formation of hetero-aggregates between microplastics (PS and PLA) and *Scenedesmus abundans*. However, in the present study, the EPS seemed to impede the hetero-aggregation between the PLA NPs and algal cells. This suggests that during the hetero-aggregation between NPs and algal cells, the role of EPS is complicated. As shown in Figure 2, a substantial proportion of LB-EPS was observed after the co-settling with algal cells in the PLA NPs treatment. The result implied that the PLA NPs could readily be adsorbed onto LB-EPS via polar group interactions, leading to their detachment from the algal cell surface. Even for the PLA NPs adsorbed onto TB-EPS, their aggregation with algal cells would be limited because the TB-EPS may favor repulsive interactions through steric hindrance effects [49]. With respect to the PS NPs in our study, the EPS seemed to promote hetero-aggregation with algal cells. The result can be explained by the secretion of TB-EPS, which was more overwhelming than the LB-EPS during the aggregation. In this context, the TB-EPS would be the main interface for PS NPs to interact with the algal cells. Moreover, compared to PLA NPs, PS NPs may be more likely to permeate into the EPS matrix via hydrophobic interactions, thereby lowering the steric resistance [50,51]. Collectively, the primary hetero-aggregation with different types of NPs would trigger stress effects and stimulate *C. vulgaris* to secrete various fractions of EPS, which in turn would mediate the hetero-aggregation process in different ways.

### 3.3. NP Toxicity to Algae

The findings from the TEM observation, DLVO calculation, and settling experiments, together with the EPS analyses in our study, have demonstrated that PS and PLA NPs could aggregate with *C. vulgaris*, and that this hetero-aggregation process was associated with EPS mediation. As a result, the stress effects of NPs on algal cells would be triggered and affected by the interactions during the hetero-aggregation, leading to complicated toxicity outcomes. Hence, various toxicity endpoints of *C. vulgaris* under different NP exposures and EPS conditions were further examined and analyzed in the present study.

#### 3.3.1. Effects of NPs on Algal Growth

**Figure 3:**
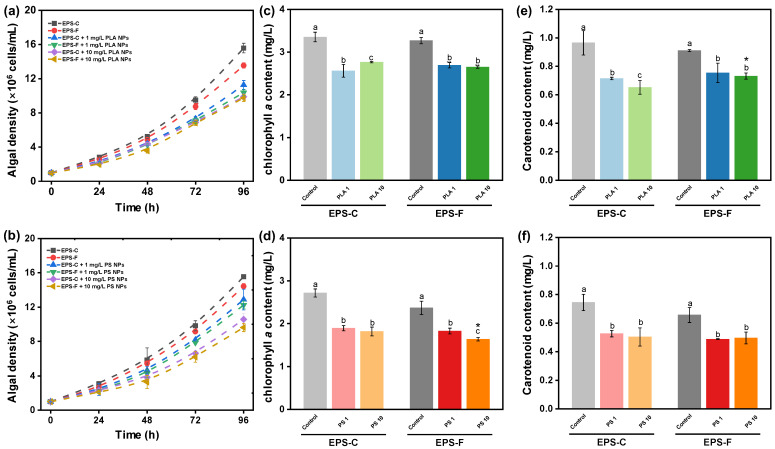
The growth curves of *C. vulgaris* when exposed to PLA (**a**) and PS NPs (**b**) in different EPS conditions for 96 h. Changes in algal chlorophyll a contents after 96 h PLA (**c**) and PS NP (**d**) exposure in different EPS conditions. Changes in algal carotenoid contents after 96 h PLA (**e**) and PS NP (**f**) exposure in different EPS conditions. Different lowercase letters indicate significant differences among groups at different NP concentrations within the EPS-C or EPS-F condition (*p* < 0.05). Asterisks in the EPS-F condition indicate values that are significantly different from those corresponding values in the EPS-C condition (*p* < 0.05).

The growth inhibitions of *C. vulgaris* exposed to different concentrations of PLA and PS NPs are shown in Figure 3a,b. After 96 h incubation, the control levels of algal density were (15.6 ± 0.56) (EPS-C) and (13.6 ± 0.31) × 10^6^ cells/mL (EPS-F) for PLA NPs, and were (15.5 ± 0.13) (EPS-C) and (14.4 ± 0.33) × 10^6^ cells/mL (EPS-F) for PS NPs. Compared to the controls, the average growth rates of *C. vulgaris* after 96 h of PS NP exposure were inhibited by 16.9% (EPS-C + 1 mg/L), 21.2% (EPS-F + 1 mg/L), 31.9% (EPS-C + 10 mg/L), and 38.1% (EPS-F + 10 mg/L), and those of PLA NP exposures were inhibited by 27.7% (EPS-C + 1 mg/L), 33.4% (EPS-F + 1 mg/L), 36.6% (EPS-C + 10 mg/L), and 37.5% (EPS-F + 10 mg/L). Both PS and PLA NPs displayed clear inhibitory effects on the growth of *C. vulgaris* within 96 h of exposure (*p* < 0.05), and the inhibitions were concentration dependent. As the occurrence of hetero-aggregation between NPs and algal cells has been demonstrated from the aforementioned results, the growth inhibition of *C. vulgaris* could be explained by the shading effects due to the formation of hetero-aggregates [52]. The shading effects would inhibit the utilization of light energy in the photochemical electron transport system, thereby reducing nutrient and gas exchanges and suppressing algal growth [9,53]. As observed in the TEM images in Figure 1a–d, hetero-aggregation was able to attract and retain more NPs around the algae, which further corroborated the shading effects. Moreover, the cell deformation and NP intrusion were observed in the TEM images, which may trigger the overproduction of ROS [54]. ROS generation could damage cellular structure, disrupt cell signaling, and inhibit algal growth [55].

In this study, the EPS condition (i.e., EPS-C and EPS-F) did not induce a significant difference in algal growth under the exposure to NPs (*p* > 0.05), suggesting the EPS barrier could not completely prevent or counteract the effect of NP stress on algal growth. EPS secretion acts as an adaptive mechanism and is essential for metabolic processes, survival capabilities, and cellular adaptation in algal cells [56]. Previous studies have suggested that EPS might serve as a physical barrier that inhibits the direct interaction between algal cells and nanoparticles [26,57,58]. However, apart from hetero-aggregation, the shading effect from NPs can be induced without direct interaction, as light attenuation would occur in a non-contact condition [59]. This may explain the limited role of EPS in the growth inhibition of *C. vulgaris* in the present study.

#### 3.3.2. Effects of NPs on Algal Pigment Synthesis

Variations in the photosynthetic pigment contents of *C. vulgaris* exposed to different concentrations of PLA and PS NPs for 96 h are presented in Figure 3. The chlorophyll a contents of *C. vulgaris* after 96 h in the controls for PLA NPs were 3.4 ± 0.11 and 3.3 ± 0.07 mg/L in EPS-C and EPS-F conditions, respectively, and those under NP exposures were inhibited by 23.6% (EPS-C + 1 mg/L), 17.7% (EPS-F + 1 mg/L), 17.6% (EPS-C + 10 mg/L), and 19.0% (EPS-F + 10 mg/L). In the controls for PS NPs, the chlorophyll a contents were 2.7 ± 0.09 and 2.4 ± 0.16 mg/L in EPS-C and EPS-F conditions, respectively. Compared to the controls, the chlorophyll a contents after 96 h of PS NP exposure were inhibited by 30.3% (EPS-C + 1 mg/L), 22.9% (EPS-F + 1 mg/L), 33.1% (EPS-C + 10 mg/L), and 31.0% (EPS-F + 10 mg/L) (Figure 3c,d). For the carotenoids, the control levels were 1.0 ± 0.09 (EPS-C) and 0.9 ± 0.01 mg/L (EPS-F) for PLA NPs, and were 0.7 ± 0.06 (EPS-C) and 0.7 ± 0.05 mg/L (EPS-F) for PS NPs. The inhibition rates were 25.9% (EPS-C + 1 mg/L), 17.3% (EPS-F + 1 mg/L), 32.4% (EPS-C + 10 mg/L), and 19.7% (EPS-F + 10 mg/L) under PLA NPs exposures, and were 29.4% (EPS-C + 1 mg/L), 25.8% (EPS-F + 1 mg/L), 32.5% (EPS-C + 10 mg/L), and 24.6% (EPS-F + 10 mg/L) under PS NPs exposures (Figure 3e,f). The inhibitions in the contents of chlorophyll a and carotenoids revealed that the exposure to NPs had a negative impact on algal pigment synthesis. It has been reported that the shading effects and cell intrusion of NPs could impede algae photosynthesis by disturbing the reaction center of the photosystem, the position of the electron donor, and the electron transport chain [24]. In the present study, the different EPS conditions did not lead to obvious changes in algal pigment synthesis under NP exposures in most cases (*p* > 0.05), which was similar to the results for growth inhibition. Pigment synthesis plays a critical role in algae photosynthesis and growth. Hence, the coherent results of the insignificant EPS influences between pigment synthesis and growth can be expected. This further supported the idea that the non-contact shading of NPs could elicit toxicity to algae, bypassing the EPS barrier.

#### 3.3.3. Effects of NPs on Algal Membrane Integrity

**Figure 4:**
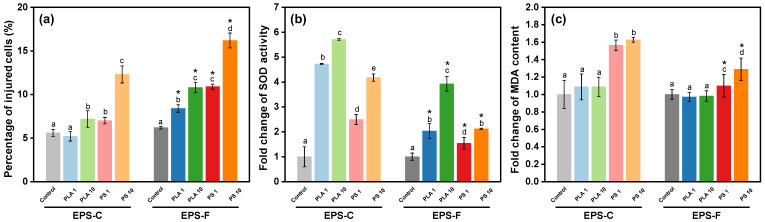
Changes in cell membrane damage (**a**), SOD activity (**b**), and MDA content (**c**) after 96 h PLA and PS NP exposures in different EPS conditions. Different lowercase letters indicate significant differences among groups at different NP concentrations within the EPS-C or EPS-F condition (*p* < 0.05). Asterisks in the EPS-F condition indicate values that are significantly different from those corresponding values in the EPS-C condition (*p* < 0.05).

Changes in the membrane integrity of *C. vulgaris* across different treatments are presented in Figure 4a. After 96 h of NP exposure, decreases in membrane integrity can be found in all the treatments. By quantifying the PI fluorescence intensity, the percentages of injured cells in most of the NP treatments were higher than those in the controls, with the highest percentage of 16.2 ± 0.85% observed in the treatment of 10 mg/L PS NPs and EPS-F condition. The results corroborated the TEM observations of NP intrusion into algal cells. Once forming hetero-aggregates, the algal membrane would be the primary interface for interaction between NPs and algal cells. We found that exposures to PS NPs resulted in higher percentages of injured cells than PLA NPs (*p* < 0.05), which were in harmony with the hetero-aggregation behaviors. Generally, endocytosis and passive diffusion are considered the main pathways for NP intrusion into algal cells [60], and both the intrusion pathways are contact processes [61], implying that the hetero-aggregation could be beneficial for the NP intrusion into algal cells. The results suggested that apart from non-contact shading effects, NPs may intrude into algal cells after hetero-aggregation, potentially inducing ultrastructure damage and toxicological effects. Moreover, the percentages of injured cells in the EPS-C conditions were generally lower than those in the EPS-F conditions (*p* < 0.05) (Figure 4a), despite the elevated hetero-aggregation efficiency of PS NPs with algae in the EPS-C condition. The result suggested that although the EPS favored the hetero-aggregation between PS NPs and algal cells, it may protect the algal cells from the intrusion of PS NPs to a certain degree.

#### 3.3.4. Effects of NPs on Algal Antioxidant System

Changes in the oxidative stress indices of algae across different treatments are depicted in Figure 4. When exposed to PLA NPs, the increased fold-changes in SOD activities compared to the control were 3.7 ± 0.02 (EPS-C + 1 mg/L), 1.0 ± 0.30 (EPS-F + 1 mg/L), 4.7 ± 0.03 (EPS-C + 10 mg/L), and 2.9 ± 0.30 (EPS-F + 10 mg/L). Exposure to PS NPs induced increased fold-changes of 1.5± 0.20 (EPS-C + 1 mg/L), 1.5 ± 0.24 (EPS-F + 1 mg/L), 3.2 ± 0.15 (EPS-C + 10 mg/L), and 1.1 ± 0.03 (EPS-F + 10 mg/L) in SOD activity (Figure 4b). Overall, NP treated cells showed significantly higher SOD activity than the control (*p* < 0.05). The observed phenomenon may be ascribed to the activation of the algal antioxidant system, which was stimulated by the heightened activity of enzymes like SOD to scavenge ROS in response to NP stress [55]. For MDA content, compared to the control, PS NP treatments displayed substantial elevations in most cases, showing increased fold-changes of 0.6 ± 0.06 (EPS-C + 1 mg/L), 0.1 ± 0.13 (EPS-F + 1 mg/L), 0.6 ± 0.03 (EPS-C + 10 mg/L), and 0.3 ± 0.13 (EPS-F + 10 mg/L). However, PLA NP treatments exhibited negligible effects on MDA contents (*p* > 0.05) (Figure 4c).

As a by-product, MDA content reflects the level of lipid peroxidation [62]. In the present study, the elevated SOD activities combined with insignificant changes in MDA contents suggested that *C. vulgaris* could effectively withstand oxidative stress of PLA NP by enhancing the activity of antioxidant defense enzymes. In contrast, the higher MDA contents induced by PS NPs indicated that the antioxidant defense enzymes of *C. vulgaris* could not cope with the oxidative stress of PS NPs, and the lipid peroxidation consequently occurred. Likewise, Li et al. [63] found that the MDA contents of marine algae (*Skeletonema costatum*) increased with increasing concentrations of PS microplastics, whereas the MDA contents under PLA microplastic exposures were not significantly changed. In previous studies, the hypotoxicity of PLA to algae was generally interpreted as its biodegradability, which could be assimilated by algae as a carbon source and favor the growth of the algae [64]. Here, by analyzing the interactive behaviors between NPs and algal cells, we consider that the low oxidative stress of PLA NPs could also be attributed to the lower hetero-aggregation efficiency compared to PS NPs and thus the contact toxicity.

As a protecting layer outside the algal cells, algal-derived EPS is generally considered to be able to mitigate the oxidative toxicity from nanoparticles [65,66]. In our study, both the oxidative effects of PS and PLA NPs were affected by EPS conditions. But, we noticed that the affection patterns of EPS were different between PS and PLA NPs. For PLA NPs, the EPS-C treatment showed a similar MDA content to the EPF-F treatment (*p* > 0.05), suggesting a potential protective effect of EPS to oxidative stress from PLA NPs. In contrast, under the PS NPs stress, both the SOD activity and MDA content were higher in the EPS-C treatment compared to those in the EPS-F treatment (*p* < 0.05). Counterintuitively, it seemed that the algal-derived EPS was more of an exacerbator than a palliative for the oxidative toxicity of PS NPs. Taking the co-settling results into account, we found that the complicated role of EPS in mediating the oxidative stress of NPs was in harmony with the hetero-aggregation behavior in the present study. As mentioned above, the EPS could promote the hetero-aggregation of algal cells with PS NPs and was unfavorable for that of PLA NPs. This may correspondingly intensify the oxidative stress of PS NPs and reduce that of PLA NPs. Our results suggest that algal-derived EPS cannot be simply considered as a counteractor to the oxidative stress of NPs. Based on the specific types of NPs, the hetero-aggregation behavior regulated by EPS would be variable. This issue should be considered in evaluating the oxidative stress of different NPs on algae.

#### 3.3.5. Integrated Biomarker Response

**Figure 5:**
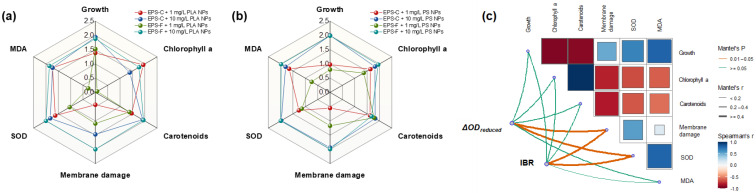
IBR plots of *C. vulgaris* after 96 h PLA (**a**) and PS NP (**b**) exposures in different EPS conditions. Mantel tests of ΔOD_reduced_ values in the co-settling experiments and IBR values in the toxicological experiments with different physiological and biochemical endpoints (**c**).

The transformed data from the toxicological endpoints in *C. vulgaris* after 96 h of exposure to PLA and PS NPs are shown in Figure 5a,b. The IBR values of *C. vulgaris* in the PS NP treatments were 13.2 (EPS-C + 1 mg/L), 12.8 (EPS-F + 1 mg/L), 15.2 (EPS-C + 10 mg/L), and 15.9 (EPS-F + 10 mg/L). They were higher than those in the PLA NP treatments, where the IBR values were 3.4 (EPS-C + 1 mg/L), 3.2 (EPS-F + 1 mg/L), 4.1 (EPS-C + 10 mg/L), and 4.1 (EPS-F + 10 mg/L). The results of IBR analysis suggested that the stress of PS NPs on *C. vulgaris* is more severe than that of PLA NPs. Previous studies generally considered that the higher stress of PS NPs may be due to the benzene ring in the PS structure, as the aromatic hydrocarbons are more reactive and have a higher toxic potential than the non-aromatic and biodegradable PLA NPs [43,64]. Based on the results from the present study, we propose another explanation from the perspective of aggregation, that compared to PLA NPs, PS NPs may be more likely to aggregate with algal cells and consequently elicit higher toxic effects on the algae. Considering that PS and PLA NPs with identical particle sizes likely possess similar shading properties, the differential IBR values observed between them suggest that the toxicity may primarily originate from contact-dependent damage associated with hetero-aggregation, rather than from a non-specific shading effect. This interpretation is further supported by the distinct hetero-aggregation behaviors of PS and PLA with algal cells, as detailed above. In future studies, the shading effect should be controlled for with an inert particle control to precisely delineate its contribution from the inherent toxicity of NPs. Moreover, potential confounding factors associated with the NPs, such as added additive antimicrobials or surfactants, could theoretically contribute to the toxicity of NPs. In the present study, the distinct toxic responses observed between PS and PLA NPs suggested that a common chemical additive was not the primary driver. This material-specific toxicity indicates that the NPs themselves were the main cause. Although the manufacturers state the absence of added additive antimicrobials or surfactants during the synthesis process of the NPs used, future work incorporating a separate leachate experiment would be valuable to assess the potential contribution from soluble additives [67]. Regardless, the results in our study indicate that when evaluating the toxicity on algae, the existing state of NPs plays an important role and should not be ignored. Likewise, Huang et al. [68] found that with a certain range of concentration and size, the density of different plastic particles was the primary factor determining their toxicity to the marine algae (*Gymnodinium aeruginosum*); those authors considered that variation in density would affect the settling and suspension states of plastic particles in seawater, and thus the probability of particles being in contact with algae cells.

In the present study, the IBR values across the treatments did not show obvious dependence on the EPS conditions, although the oxidative stress indicators (i.e., SOD and MDA) exposed to NPs were significantly affected by the EPS conditions in most cases. By screening the measured endpoints, we consider that the potential changes in IBR values may be masked by the growth and photosynthesis indicators, as the growth inhibition rates and pigment contents of *C. vulgaris* did not vary with the EPS conditions. The results further demonstrate that the general toxicity of NPs towards algae may not display a simple monotonic correlation with EPS. On one hand, the EPS could affect the contact toxicity with a certain range of NP concentrations. On the other hand, its counteraction to the non-contact toxicity, such as shading effect, may be limited.

The correlations of toxicological endpoints with hetero-aggregation (ΔOD_reduced_) and IBR are assessed by mantel tests (Figure 5c). The ΔOD_reduced_ showed significantly positive associations with membrane damage (r = 0.464, *p* < 0.05) and SOD activity (r = 0.714, *p* < 0.05). Also, the membrane damage (r = 0.585, *p* < 0.05) and SOD activity (r = 0.461, *p* < 0.05) presented significantly positive associations with IBR value. The results revealed that both the hetero-aggregation and general toxicity were positively related to cellular membrane damage and antioxidant activity, demonstrating a view that the hetero-aggregation behavior could exacerbate the contact-dependent damage and thus the general toxicity of NPs on algae, mainly through affecting the cellular membrane integrity and oxidative stress.

## 4. Conclusions

In this study, the hetero-aggregation between different types of NPs and *C. vulgaris*, as well as the toxicological consequences, were investigated. The potential roles of EPS in these interaction processes were also evaluated. Our results showed that PS NPs had higher agglomeration efficiencies with *C. vulgaris* than PLA NPs, which were inconsistent with DLVO theory and empirical data from microplastic studies. This could be explained by the fact that aromatic PS NPs triggered stronger self-protective feedback in *C. vulgaris* and stimulated the secretion of protein-enriched EPS, especially TB-EPS, which acted as binders to enhance the formation of hetero-aggregation. The enhanced hetero-aggregation of PS NPs could, in turn, potentiate the toxicity, mainly by mediating contact-dependent damage, such as cellular membrane integrity and oxidative stress. The present study underscores the material-specific uniqueness of NPs in interactions with freshwater algae. Further studies are needed to broaden our knowledge of the hetero-aggregation behaviors and toxicological effects of NPs, particularly the divergent roles of EPS in mediating the interactive effects of different types of NPs on freshwater algae.

## Figures and Tables

**Table 1:** Fitting parameters for co- and additive-settling curves of PS and PLA NPs with *C. vulgaris* under different EPS conditions.

NPs	EPS Condition		*OD_plateau_*	*OD_reduced_*	*v*	*R* ^2^	Δ*OD_reduced_*
PS	EPS-C	Sum	−0.076	1.076	0.037	0.998	0.74
		Mix	−0.818	1.817	0.033	0.998	
	EPS-F	Sum	−0.227	1.227	0.041	0.998	0.53
		Mix	−0.758	1.758	0.031	0.997	
PLA	EPS-C	Sum	−0.071	1.071	0.037	0.998	0.33
		Mix	−0.404	1.404	0.043	0.997	
	EPS-F	Sum	−0.174	1.174	0.044	0.998	0.49
		Mix	−0.669	1.669	0.033	0.991	

## Data Availability

The original contributions presented in this study are included in the article. Further inquiries can be directed to the corresponding authors.

## Supplementary Materials

The following supporting information can be downloaded at https://www.mdpi.com/article/10.3390/toxics13110980/s1: Figure S1: Representative TEM images of EPS-C (a) and EPS-F (b) algal cells; Figure S2: Microscope images of the algal cells before (a) and after (b) sonication at 40 kHz for 5 min at 25 °C; Figure S3: Size distribution of the original PLA (a) and PS NPs (b) analyzed by ImageJ (1.54) (n = 60 for each plastic); Figure S4: FTIR images of the original PLA (a) and PS NPs (b); Figure S5: Individual settling curves of PS NPs, PLA NPs, EPS-C algae, and EPS-F algae; Table S1: The culture media components of BG-11; Table S2: The composition of A5 solution; Table S3: Zeta potential and size of algae cells before settling experiment; Table S4: Readings at different wavelengths in spectrometry-based assays of pigment and oxidative stress markers among the treatments of NP-free, PS NP-added, and PLA NP-added; Text S1: NP exposures [69,70,71,72,73].
