# Supplementary material for: Hetero-Aggregation of Nanoplastics with Freshwater Algae and the Toxicological Consequences: The Role of Extracellular Polymeric Substances
Source: Toxics. 2025 Nov 14;13(11):980. doi: 10.3390/toxics13110980 (PMC12656403; doi:10.3390/toxics13110980)
Supplement: Supplementary file 1 [file toxics-13-00980-s001.zip › toxics-3979735-supplementary.pdf]

## -Supplementary Materials for

**Hetero-Aggregation of Nanoplastics with Freshwater Algae and the Toxicological Consequences: The Role of Extracellular Polymeric Substances**

Jiannan Ding [a,b,c], Jiaxin Yang [a], Xiaojun Song [a*], Shuo Liu [a], Zhenguo Wang [a], Hua Zou [a,b,c*]

[a] *School of Environment & Ecology, Jiangnan University, Wuxi, 214122, China*

[b] *Jiangsu Collaborative Innovation Center of Technology and Material of Water Treatment, Suzhou, 215009, China*

[c] *Biomass Energy and Biological Carbon Reduction Engineering Center of Jiangsu Province, Wuxi, 214122, China*

* Correspondence to: Xiaojun Song (7201403003@stu.jiangnan.edu.cn);

Hua Zou (zouhua@jiangnan.edu.cn)

## List of Supplementary Materials

## Materials and Methods:

**Text S1.** NP exposures

## List of Tables:

**Table S1.** The culture media components of BG-11.

**Table S2.** The composition of A5 solution.

**Table S3.** Zeta potential and size of algae cells before settling experiment.

**Table S4.** Readings at different wavelengths in spectrometry-based assays of pigment and oxidative stress markers among the treatments of NP-free, PS NP-added, and PLA NP-added.

## List of Figures:

**Figure S1.** Representative TEM images of EPS-C (a) and EPS-F (b) algal cells.

**Figure S2.** Microscope images of the algal cells before (a) and after (b) sonication at 40 kHz for 5 min at 25 °C.

**Figure S3.** Size distribution of the original PLA (a) and PS NPs (b), analyzed by ImageJ (n = 60 for each plastic).

**Figure S4.** FTIR images of the original PLA (a) and PS NPs (b).

**Figure S5.** Individual settling curves of PS NPs, PLA NPs, EPS-C algae, and EPS-F algae.

## 1. Text S1: NP exposures

In natural aquatic systems, for example, microplastic (totally defined as plastic particles < 5 mm) concentrations ranged from 0 to $3.8 \times 10^{-3}$ mg/L across four estuarine rivers in the Chesapeake Bay (Yonkos et al., 2014) and from 0 to $7.0 \times 10^{-1}$ mg/L in the Danube River (Lechner et al., 2014). The highest abundance of microplastics in freshwater systems globally has been noted in Taihu Lake, China, with levels ranging from 30 to 50 mg/L (Su et al., 2016). Nevertheless, numerous studies that assess the effects of micro- and nanoplastics on biochemical processes have employed particle concentrations significantly higher than those typically found in natural environments, occasionally reaching levels between 250 and 1000 mg/L (Besseling et al., 2014; Sjollema et al., 2016). In this context, this methodology may result in a misinterpretation of the environmental influence of NPs. Therefore, in this study, we used polylactic acid (PLA) and polystyrene (PS) nanoparticles at environmentally relevant concentrations of 1 and 10 mg/L to investigate their potential toxicity towards microalgae.

Table S1. The culture media components of BG-11.

| Compound | g/L |
|---|---|
| $NaNO_3$ | 1.500 |
| $K_2HPO_4$ | 0.040 |
| $MgSO_4 \cdot 7H_2O$ | 0.075 |
| $CaCl_2 \cdot 2H_2O$ | 0.036 |
| Citric acid | 0.006 |
| $Na_2$ EDTA | 0.001 |
| $Na_2CO_3$ | 0.020 |
| Ferric Ammonium citrate | 0.006 |
| A5 solution | 1.000 mL |

Table S2. The composition of A5 solution.

| Compound | g/100 mL |
| --- | --- |
| $H_3BO_3$ | 0.286 |
| $MnCl_2 \cdot 4H_2O$ | 0.186 |
| $ZnSO_4 \cdot 7H_2O$ | 0.022 |
| $Na_2MoO_4 \cdot 2H_2O$ | 0.039 |
| $CuSO_4 \cdot 5H_2O$ | 0.008 |
| $Co(NO_3)_2 \cdot 6H_2O$ | 0.005 |

Table S3. Zeta potential and size of algae cells before settling experiment.

| Exposure condition | Zeta potential (mV) | Size (nm) |
|---|---|---|
| EPS-C *C. vulgaris* | -24.00 ± 0.78 | 7913.33 ± 835.17 |
| EPS-F *C. vulgaris* | -20.07 ± 0.49 | 6307.67 ± 202.35 |

Table S4. Readings at different wavelengths in spectrometry-based assays of pigment and oxidative stress markers among the treatments of NP-free, PS NP-added, and PLA NP-added.

| Treatment | Pigment | | | Oxidative stress markers | | |
|---|---|---|---|---|---|---|
| | $A_{470}$ | $A_{652}$ | $A_{665}$ | $A_{450}$ | $A_{532}$ | $A_{600}$ |
| NP-free | 0.028 ± 0.003 | 0.014 ± 0.001 | 0.025 ± 0.006 | 1.303 ± 0.008 | 0.023 ± 0.005 | 0.001 ± 0.000 |
| PS NP-added | 0.028 ± 0.005 | 0.015 ± 0.003 | 0.024 ± 0.002 | 1.298 ± 0.015 | 0.022 ± 0.006 | 0.001 ± 0.000 |
| PLA NP-added | 0.027 ± 0.004 | 0.015 ± 0.002 | 0.026 ± 0.004 | 1.306 ± 0.011 | 0.022 ± 0.003 | 0.001 ± 0.000 |

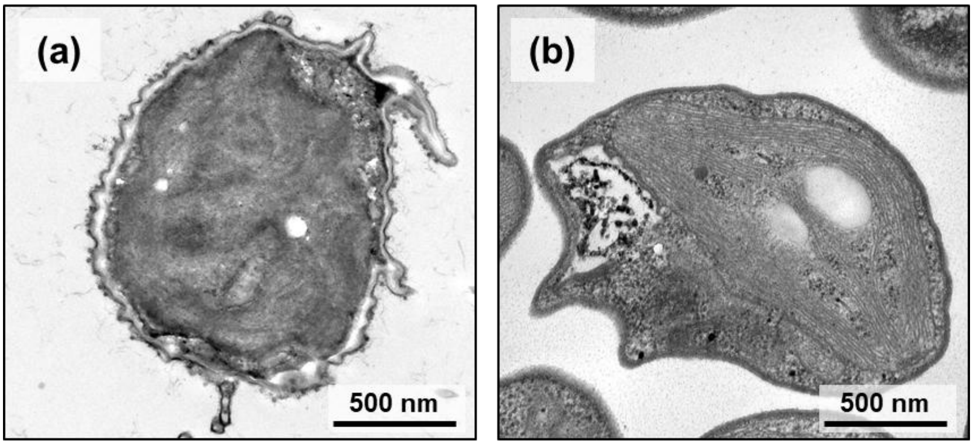


Figure S1. Representative TEM images of EPS-C (a) and EPS-F (b) algal cells.

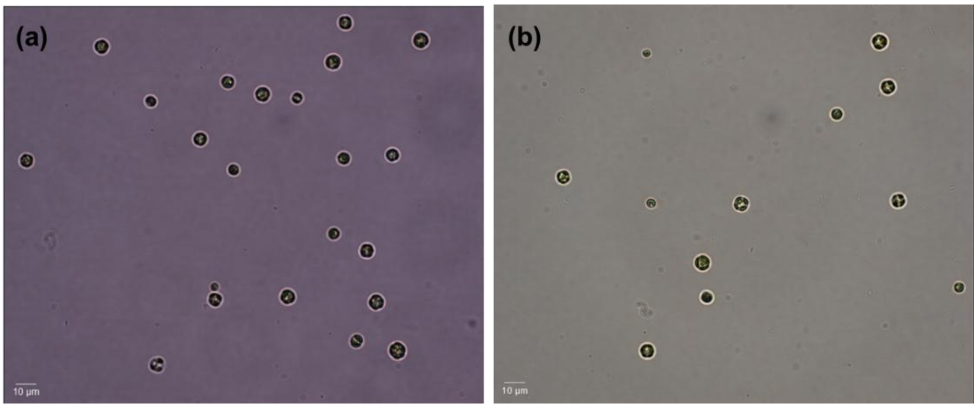


Figure S2. Microscope images of the algal cells before (a) and after (b) sonication at 40 kHz for 5 min at 25 °C.

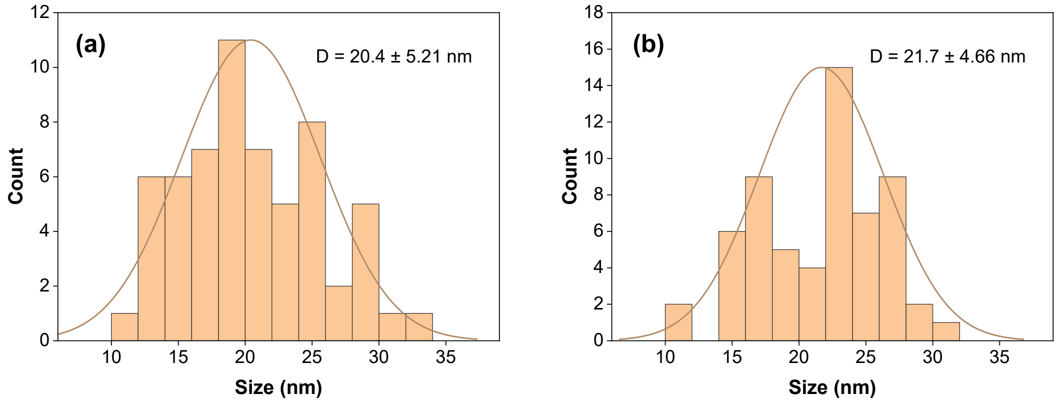


Figure S3. Size distribution of the original PLA (a) and PS NPs (b), analyzed by ImageJ (*n* = 60 for each plastic).

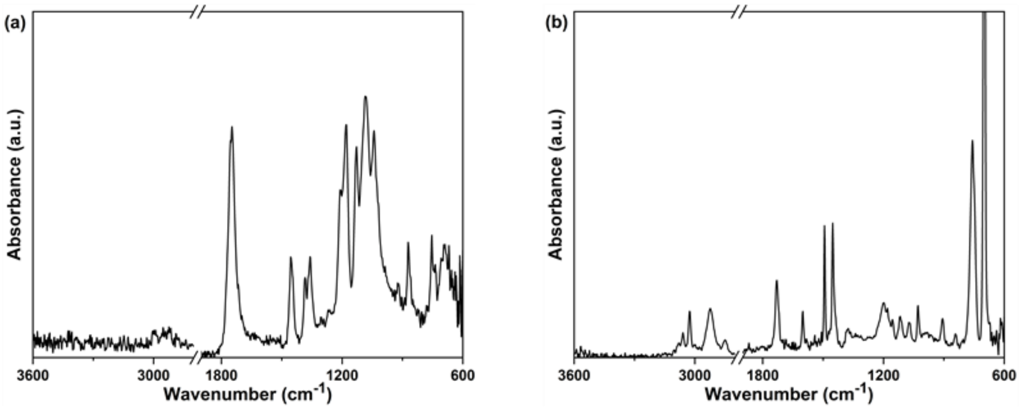


Figure S4. FTIR images of the original PLA (a) and PS NPs (b).

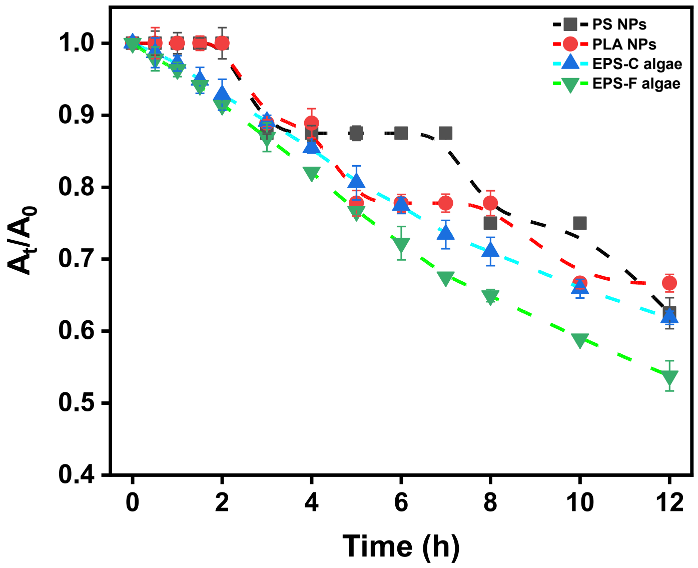


Figure S5. Individual settling curves of PS NPs, PLA NPs, EPS-C algae, and EPS-F algae.

**References:**


Besseling, E., Wang, B., Lürling, M., Koelmans, A.A., 2014. Nanoplastic affects growth of *S. obliquus* and reproduction of *D. magna*. Environ. Sci. Technol. 48(20), 12336-12343.

Lechner, A., Keckeis, H., Lumesberger-Loisl, F., Zens, B., Krusch, R., Tritthart, M., Glas, M., Schludermann, E., 2014. The Danube so colourful: A potpourri of plastic litter outnumbers fish larvae in Europe's second largest river. Environ. Pollut. 188, 177-181.

Sjollema, S.B., Redondo-Hasselerharm, P., Leslie, H.A., Kraak, M.H.S., Vethaak, A.D., 2016. Do plastic particles affect microalgal photosynthesis and growth? Aquat. Toxicol. 170, 259-261.

Su, L., Xue, Y., Li, L., Yang, D., Kolandhasamy, P., Li, D., Shi, H., 2016. Microplastics in Taihu Lake, China. Environ. Pollut. 216, 711-719.

Yonkos, L.T., Friedel, E.A., Perez-Reyes, A.C., Ghosal, S., Arthur, C.D., 2014. Microplastics in four estuarine rivers in the Chesapeake Bay, U.S.A. Environ. Sci. Technol. 48(24), 14195-14202.
